# Association between pesticide exposure and neurobehavioral performance of agricultural workers: A cross‐sectional study

**DOI:** 10.1002/brb3.2641

**Published:** 2022-06-06

**Authors:** Sujan Tiwari, Namrata Sapkota, Sagun Tiwari, Bhanu Sapkota

**Affiliations:** ^1^ Institute of Agriculture and Animal Science Tribhuvan University Kathmandu Nepal; ^2^ Net Fresh Hospital Chitwan Nepal; ^3^ Om Wellness Hospital Biratnagar Nepal; ^4^ Shenzhen Institute of Advanced Technology, Chinese Academy of Sciences Shenzhen China; ^5^ University of Chinese Academy of Science Beijing China; ^6^ Department of Neurology and Rehabilitation Seventh People's Hospital of Shanghai University of TCM Shanghai P. R. China; ^7^ Shanghai University of TCM Shanghai P. R. China; ^8^ Life Care Hospital Chitwan Nepal; ^9^ Armed Forces Medical College Dhaka Bangladesh

**Keywords:** agriculture worker, cognitive function, farmers, neurobehavioral function, pesticide

## Abstract

**Background:**

Pesticide exposure has affected humans’ health, especially those directly in contact with a pesticide like agricultural workers. Here, we sought to see a link between pesticide exposure and cognitive impairment among male agricultural workers.

**Methods:**

We selected 18–60 years old 100 male agricultural workers who came for their health check‐up in the hospital's out‐patient department from August 2020 to May 2021. Standardized questionnaires (German Q18 questionnaire) and WHO Neurobehavioral Core Test Battery were used to examine the neurobehavioral performance of the individuals. The data were presented in a descriptive manner and statistically examined using the chi‐square test.

**Results:**

The male agricultural workers’ neurobehavioral performance was 46% normal and 54% abnormal. Headaches, impaired focus, short memory, weariness, palpitation, and numbness were the most prevalent neurobehavioral impairments. The chi‐square test revealed that spraying frequency (*p =*.010), personal protective equipment (PPE) use (*p *< .001), and smoking habits (*p =*.006) were all linked to neurobehavioral performance, while spraying duration (*p =*.804), working years (*p =*.234), pesticide types (*p =*.816), and spraying time (*p =*.867) were not linked to neurobehavioral performance.

**Conclusion:**

We found that pesticide exposure has a certain role on neurobehavioral performance in adult male agricultural workers as more people have been affected; however, a considerable number of confounding factors might have played a significant role in giving rise to such results. The government should be concerned about farmer's neurobehavioral performance and implement different approaches to minimize the use of pesticides so that agricultural workers can work without any mental health problems.

## Graphical abstract

Pesticide exposure has a certain role to play on neurobehavioral performance in adult male agricultural workers as more people have been affected; however, a considerable amount of confounding factors might have played a significant role in giving rise to such results. The government should concern about farmer's neurobehavioral performance and implement different approaches to minimize the use of pesticides so that agricultural workers can work without any mental health problems.

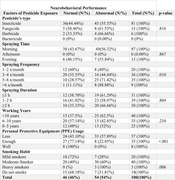

## 1 INTRODUCTION

Pesticides are commonly used as toxic chemicals in agriculture‐related fields to kill various kinds of pests (insects, fungi, microorganisms, etc.) and are thus used to enhance crops, livestock, and farm productivity. Pesticides have various adverse effects on the health of the individuals whether exposed for an extended period or short time, which could lead to numerous diseases such as cancers, asthma, allergies, hypertension, neurological disease, psychiatric disease, and even pregnancy‐related issues (low birth weight, deformities, and abortions) (Forté et al., 2021; Hongsibsong et al., 2017; Kaur & Kaur, 2018; Kim et al., 2019).

Agriculture workers use pesticides to improve their crop production; therefore, they are one of the highly exposed groups of people. Several studies have recently shown that this group has a higher prevalence rate for neurobehavioral impairment than the nonexposed (Campos et al., 2016; Faria et al., 2014; Ismail et al., 2012; Ramírez‐Santana et al., 2020). Pesticide exposure has been documented to affect motor speed and coordination, sustained attention, visuospatial ability, visual memory, and information processing speed in agricultural related workers (Ismail et al., 2017; Muñoz‐Quezada et al., 2016; Perry et al., 2020; Richardson et al., 2019; Saeedi Saravi & Dehpour, 2016; Sánchez‐Santed & Colomina, 2016). Additionally, insecticide exposure may result in altered neurobehavioral/neurotoxic effects due to cholinesterase inhibition and/or ion channel changes, and herbicide and fungicide exposure cause cytotoxicity by producing free radicals and altering mitochondrial function, respectively (Perry et al., 2020; Richardson et al., 2019; Saeedi Saravi & Dehpour, 2016; Sánchez‐Santed & Colomina, 2016). People who do agriculture‐field work themselves are from low socioeconomic status, and on top of it, if they have altered cognitive functions because of their daily routine on the field, their quality of life would be miserable and it would be difficult for them to survive from a financial crisis. Most developing nations rely on their agricultural resources for their economy, and developing nation's agriculture system might slowly fade away, impacting nation's economic growth because of their agriculture workers’ health issues. If farmers in Nepal, one of the developing and agriculture‐based countries, suffer from cognitive debility, it would directly impact the socioeconomic condition of the country. Therefore, the effect of pesticide exposure on agriculture workers’ neurobehavioral function should be a prime concern to be solved. However, based on our knowledge, no study has been done in Nepal on the impact of pesticide use on the neurobehavioral functions of agriculture workers. Thus, we aim to investigate the relationship between pesticide exposure and the neurobehavioral performance of agricultural workers residing in Nepal.

## 2 METHODS

### 2.1 Study population

Our cross‐sectional study of pesticide exposures was conducted on 18–60 years old 100 participants who came to the hospital for their health check‐up in the out‐patient department from August 2020 to May 2021 and were selected based on the inclusion and exclusion criteria (Table 1). Most of the participants were from the Chitwan district, Biratnagar District and nearby districts of those two districts of Nepal. All participants were asked to complete data collection activities and physical examination. Informed consent was obtained through face‐to‐face interviews, and trained health professionals conducted this process. The institutional review board of the hospital approved this study.

**TABLE 1:** Inclusion and exclusion criteria

Inclusion criteria	Exclusion criteria
‐ Agricultural worker/farmer ‐ Adult male 18–60 years	‐ Any neuropsychiatric disorder ‐ Any other disease that affects cognitive functions ‐ Participants using drugs that affect the central nervous system

### 2.2 Data collection

The German Q18 questionnaire was used to assess neurobehavioral performance (the questionnaire is available in English, and we translated English to the local Nepalese language orally to the participants) (Ihrig et al., 2001). It comprises 18 questions that are used to assess the symptoms of neurobehavior, in which cut‐off point 5 was implemented to determine the altered neurobehavior performance, which was recommended by a previous study to use in the German Q18 questionnaire (Ihrig et al., 2001). In addition, health professionals used the WHO Neurobehavioral Core Test Battery (WHO‐NCTB), which comprises the Digit Span, Digit Symbol, Pursuit Aiming, and Trail Making tools to assess neurobehavioral function (Anger et al., 1994). Respondents completed the test in 10 min, starting with the Digit Span Test, the Digit Symbol Test, the Pursuit Aiming Test, and finally, the Trail Making Test. If all of the score tests were over 40, the neurobehavioral performance was categorized as normal, and if one of the score tests was below 40, it was classified as abnormal (Anger et al., 1994; Ihrig et al., 2001). As our study aims to explore whether those agricultural field workers have abnormal neurobehavior function, we used the above‐mentioned cut‐off points to differentiate between normal and abnormal neurobehavior functions.

### 2.3 Statistical analysis

The data were presented in a descriptive manner using frequencies and percentage ages to show participants’ characteristics, pesticide exposure factors, and neurobehavioral function. Using chi‐square tests, the relationship between pesticide exposure and neurobehavioral performance was examined. IBM SPSS 23.0 (Chicago, IL, USA) was used to conduct all analyses, and statistical significance was defined as a *p*‐value of less than .05.

## 3 RESULTS

The group of 48 to 57 years old had the most subjects out of the four age groups, and 55% of participants had a normal nutritional status. While half of the participants were moderate smokers, three fifths of the subjects did not consume alcohol, and a very large majority of the participants (87%) were married (Table 2). The German Q18 questionnaire was used to screen for neurobehavioral problems (Table 3). Using a cut‐off value of 5, 56 people (46%) were classified as abnormal, while the rest had a normal neurobehavioral function. The most prevalent neurobehavioral deficits were short memory (55%), attention difficulties (59%), frequent headache (70%), weariness (46%), palpitation (44%), and numbness (44%).

**TABLE 2:** Basic characteristics of participants

Characteristics	Number (N)	Percentage
Age		
18−27 years	14	14
28−37 years	26	26
38−47 years	39	39
48−57 years	46	46
Nutritional status		
Underweight	15	15
Overweight	17	17
Obesity	13	13
Normal	55	55
Smoking habit		
Mild smokers	20	20
Moderate smoker	40	40
Heavy smokers	3	3
Do not smoke	18	18
Alcohol consumption		
Yes	40	40
No	60	60
Marital status		
Unmarried	9	9
Married	87	87
Divorced/widowed	4	4

**TABLE 3:** Participant's response to the German Q18 questionnaire

		Responses	
No.	Questions	Yes, N (%)	No, N (%)
1	Do you have a short memory?	55 (55)	45 (45)
2	Have your relatives told you that you have a short memory?	30 (30)	70 (70)
3	Do you often have to make notes about what you must remember?	20 (20)	80 (80)
4	Do you generally find it hard to get the meaning from reading newspapers and books?	20 (20)	80 (80)
5	Do you often have problems with concentrating?	59 (59)	41 (41)
6	Do you often feel irritated without any particular reason?	44 (44)	56 (56)
7	Do you often feel depressed without any particular reason?	10 (10)	90 (90)
8	Are you abnormally tired?	46 (46)	54 (54)
9	Do you have palpitation of the heart even when you do not exert yourself?	44 (44)	56 (56)
10	Do you sometimes feel an oppression in your chest?	4 (4)	96 (96)
11	Do you often perspire without any particular reason?	33 (33)	67 (67)
12	Do you have a headache at least once a week?	70 (70)	30 (30)
13	Are you less interested in sex than what you think is normal?	20 (20)	80 (80)
14	Do you often feel sick?	33 (33)	67 (67)
15	Do you have numb feelings in your hands or feet?	44 (44)	56 (56)
16	Is there a weak feeling in your arms or legs?	36 (36)	64 (64)
17	Do your hands tremble?	40 (40)	60 (60)
18	Does alcohol not agree with you?	80 (80)	20 (20)

In our study, WHO‐NCTB found that 46% of participants had normal neurobehavioral performance, and 54% had abnormal neurobehavioral performance, which matched the German Q18 questionnaire. The Digit Span Test, Digit Symbol Test, Pursuit Aiming Test, and Trail Making Test all showed a significant connection with the neurobehavioral performance, with *p* < .001 being the most significant (Table 4).

**TABLE 4:** Neurobehavioral performance on neurobehavioral test and its statistical analysis

	Neurobehavioral performance				
	Normal		Abnormal		
Neurobehavioral test	N	%	N	%	*p*‐Value
Digit Span	83	83	35	17	<.001
Digit Symbol	75	75	25	25	<.001
Pursuit Aiming	79	79	21	21	<.001
Trail Making	85	85	15	15	<.001
Neurobehavioral performance	47	46	53	54	

The findings of the chi‐square test were used to investigate the link between pesticide exposure parameters and farmers’ neurobehavioral performance (see Table 4). Pesticide types, spraying times, spraying duration, or working years had no significant connection with neurobehavioral performance (*p*‐values = .816, .867, .804, and .234, respectively). However, there was a correlation between neurobehavioral performance and pesticide spraying frequency (*p =*.010), personal protective equipment (*p* = .001), and smoking habit (*p =*.006) (Table 5).

**TABLE 5:** Factors of pesticide exposure type and its relation with neurobehavioral performance

Neurobehavioral performance				
Factors of pesticide exposure	Normal, N (%)	Abnormal, N (%)	Total, N (%)	*p*‐Value
Pesticide types				
Insecticide	36 (44.44)	45 (55.55)	81 (100)	
Fungicide	5 (38.46)	8 (61.53)	13 (100)	.816
Herbicide	2 (33.33)	4 (66.66)	6 (100)	
Bactericide	0 (0)	0 (0)	0 (0)	
Spraying time				
Morning	38 (43.67)	49 (56.32)	87 (100)	
Afternoon	0 (0)	0 (0)	0 (0)	.867
Evening	6 (46.15)	7 (53.84)	13 (100)	
Spraying frequency				
1−2 x/month	12 (60)	8 (40)	20 (100)	
3−4 x/month	20 (55.55)	16 (44.44)	36 (100)	.010
5−6 x/month	10 (28.57)	25 (71.42)	35 (100)	
>6 x/month	1 (11.11)	8 (88.88)	9 (100)	
Spraying duration				
≤1 h	12 (38.70)	19 (61.29)	31 (100)	
1−2 h	16 (41.02)	23 (58.97)	39 (100)	.804
≥2 h	10 (33.33)	20 (66.66)	30 (100)	
Working years				
>10 years	15 (37.5)	25 (62.5)	40 (100)	
6−10 years	20 (57.14)	15 (42.85)	35 (100)	.234
0−5 years	12 (48)	13 (52)	25 (100)	
Personal protective equipment usage				
Less	24 (43.10)	33 (57.89)	57 (100)	
Enough	27 (77.14)	8 (22.85)	35 (100)	.001
Well	8 (100)	0 (0)	8 (100)	
Smoking habit				
Mild smokers	18 (72)	7 (28)	20 (100)	
Moderate smoker	20 (40)	30 (60)	40 (100)	
Heavy smokers	0 (0)	3 (100)	3 (100)	.006
Do not smoke	15 (68.18)	7 (31.81)	18 (100)	
Total	46 (46)	54 (54)	100 (100)	

## 4 DISCUSSION

Our study showed that more than half of the recruited agricultural workers had an altered neurobehavioral function, although we could not find any correlation of pesticide types, pesticide spraying time, and working years in the agriculture field with altered neurobehavioral functions. However, there was a definite link between smoking habits, use of personal protective equipment (PPE), and pesticide spraying frequency with cognitive deficits.

Emerging studies documented that acute and chronic pesticide exposure is associated with adverse influences and hostile health conditions that directly or indirectly lead to many diseases or disorders, including neurological disorders and/or impaired neurobehavioral performance (Kamel & Hoppin, 2004; London et al., 2012; Lucero & Muñoz‐Quezada, 2021; Ramírez‐Santana et al., 2020). Although Nepal is an agriculture‐based country, few studies have been done to evaluate the effect of pesticide exposure on neurobehavioral symptoms in the agriculture worker group. This working group is one of the prime backbones for developing the nation, but whether pesticide, frequently used by most agriculture‐related personnel in Nepal, has impacted their neurobehavior and cost their health is still unknown. This study was done in the midst of the corona pandemic, and it was challenging to collect data door to door. Therefore, we recruited participants who came to the hospital, mainly from Chitwan district, Biratnagar district, and nearby districts of those two districts of Nepal, for health‐check up and excluded participants with any disorder/disease which might alter neurobehavioral functions. Based on the finding of this study, several pesticide exposure factors, such as spraying frequency, smoking habit, and personal protective equipment, are all factors to be considered, except for pesticide types, spraying duration, working years, and spraying time, which correspond with neurobehavioral performance.

In our study, one of the reasons why we have found neurobehavioral deficits is that the agricultural workers included in our study were mostly 48−57 years of age. In addition, beyond 28 years of age, nerve function declines every 5 years, with the decline in nervous system performance because the harmful chemicals from the pesticide go to the brain cells through the various biological mechanism and cause deterioration of nerve cell, leading to altered neurobehavioral functions (Jett, 2011; Rohlman et al., 2012). Studies have also reported that pesticides cause alterations in neurotransmitter systems, ion channels, mitochondrial function, cholinergic mechanism, and free radical production, which all lead to dysfunctional neurobehavior functions in individuals. In addition, respondents with abnormal nutritional status had inferior neurobehavioral performance than those with an adequate nutritional condition due to endocrine disruption (LaVerda et al., 2015).

A relationship was established between the use of personal protection equipment and neurobehavioral function in the study. The majority of responders found PPE to be ineffective. PPE is well known for limiting pesticide exposure and avoiding pesticide‐related health effects. The poor use of PPE is correlated with lack of authentic PPE availability, negative attitudes, and feeling of discomfort while wearing PPE (Ismail et al., 2017). The usage of PPE by farmers is linked to their cholinesterase levels. Spraying using complete PPE is essential to avoid pesticide exposure and is thought to reduce the risk of pesticide poisoning among farmers. Therefore, if there is any mistake while wearing PPE and spraying for a longer time, it might affect the individual's health.

There was a significant link between smoking and neurobehavioral function in our study. Cigarette smoking harms human health because it contains nicotine, which substantially impacts brain function, especially in high doses, by producing endorphins, leading to nerve‐related issues (Benowitz, 2009). The majority of respondents were moderate smokers, and along with all heavy smoker respondents, demonstrated abnormal behavior. Another study found that high levels of nicotine exposure are a risk factor for tobacco farmers’ mental health, while the impact of these exposures on farmers’ mental health is unknown. Nicotine promotes cholinergic neurotransmission by binding to the acetylcholine receptor, and the basal forebrain cholinergic systems innervate the cerebral cortex and subcortical nuclei. Changes in cholinergic neurotransmission may thus have an impact on numerous areas of the brain that are involved in cognitive processes and complicated behaviors.

One of the pesticide exposure characteristics linked to neurobehavioral performance is spraying frequency. Most farmers in this study used pesticides 3–4 to 5−6 times each month. Spraying frequency is determined by crop type, pesticide type, and pests. The higher the insect population, the more frequently pesticides are sprayed, resulting in more chemical exposure and a higher risk of neurobehavioral consequences. The spraying duration is another key pesticide exposure element linked to neurobehavioral disorders; however, our study did not show such correlation. Likewise, although working years were linked with neurological issues, the study did not have such a relationship. Altogether, spraying duration, working years, pesticide types, and spraying time pesticide exposure parameters were not linked to neurobehavioral performance, despite contradicting earlier research. Neurobehavior function is the result of all sensory, motor, and complex interconnected nervous system functions. In addition, studies have reported that various pesticides affect the individual's complex nervous system, causing cognitive deficits by hampering the brain's synapse, neurotransmitter synthesis, and other cognitive‐related processes. What is more, it gives rise to various neuropsychiatric sequelae such as depression, anxiety, Alzheimer's disease, and Parkinson's disease.

First, the main limitation of this study is that we used a structured questionnaire and self‐reporting data, making causal correlations between variables challenging to discern, and various confounding factors might have influenced our result. Studies have found that confounding factors such as age, demographic history, personal history, family or social history, alcohol consumption, pre‐existing medical conditions, and daily habits might influence studies (Wang & Cheng, 2020; Zhang et al., 2018). Second, WHO‐NCTB pointed out that people from cultures very different from those in Europe and North America may not be tested effectively. So, this conclusion could be otherwise in people with a different culture (Anger et al., 2000). Third, there was no comprehensive analysis of the pesticides used, and no quantitative biomarker measurements were taken. Fourth, we did not analyze scalar information of actual scores on the individual test.

## 5 CONCLUSION

Even though many cofounding factors might have played a role in the outcome of this study, there is undoubtedly a negative correlation between pesticide exposure and the neurobehavioral performance of adult male agricultural workers. As a corollary, the government should be vigilant about farmers’ neurobehavioral performance and pursue various strategies for reducing pesticide use so that agricultural workers can work without mental health issues. The government must also provide frequent pesticide counseling, including selecting appropriate pesticides, correctly administering them, and informing farmers about the importance of wearing PPE. In the future, pesticide exposure and psychiatric diseases should be probed further, with a detailed and quantitative investigation of the substances or mixes of pesticides implicated in an exposure. Furthermore, we suggest that psychological testing should be undertaken in pesticide‐exposed populations.

## CONFLICT OF INTEREST

The authors declare no conflict of interest.

## FUNDING INFORMATION

None.

## AUTHOR CONTRIBUTIONS

*Conception and design*: Sagun Tiwari and Sujan Tiwari. *Administrative support*: all authors. *Provision of study materials*: all authors. *Collection, assembly, and interpretation of data*: all authors. *Manuscript writing*: Sagun Tiwari and Sujan Tiwari. *Final approval of manuscript*: all authors.

## Data Availability

Data/reports are available from the authors upon reasonable request and with the participant's permission.
